# An unusual finding in a 57-year-old woman with new onset hypertension and a diastolic murmur

**DOI:** 10.1136/heartjnl-2016-309661

**Published:** 2016-07-13

**Authors:** Jason M Tarkin, James H F Rudd, David R Jayne, Rosemary A Rusk, Deepa Gopalan

**Affiliations:** 1 Division of Cardiovascular Medicine, University of Cambridge, Cambridge, UK; 2 Vasculitis and Lupus Service, Cambridge University Hospitals NHS Trust, Cambridge, UK; 3 Division of Cardiovascular Medicine, Cambridge University Hospitals and Papworth Hospital NHS Trust, Cambridge, UK; 4 Department of Radiology, Cambridge University Hospitals and Imperial College Healthcare NHS Trust, Cambridge, UK

## Abstract

**Clinical introduction:**

A 57-year-old woman presented to our clinic with breathlessness brought on while walking uphill. She had been recently diagnosed with systemic hypertension. There was no known family history of cardiac disease, or prior smoking habit. On examination, pulse was 73 bpm and blood pressure 155/73 mm Hg, which was asymmetrical in her arms. Auscultation revealed a readily audible early diastolic murmur in the aortic area and bilateral subclavian bruits. ECG showed sinus rhythm with no abnormality. Transthoracic echocardiography demonstrated mild-to-moderate aortic regurgitation, and normal left ventricular size and function. The ascending aorta was mildly dilated (41 mm), with para-aortic thickening noted. Owing to the abnormal appearance of the aortic wall, cardiac MRI, and subsequently ^18^F-fluorodeoxyglucose (FDG) positron emission tomography (PET) scan was performed (figure 1).

**Question:**

Which complication of the underlying disease is evident in figure 1, panel C?

- Aortic aneurysm
- Aortic dissection
- Aortic thrombus
- Coronary artery aneurysm
- Coronary sinus fistula

[Fig HEARTJNL2016309661F1]

**Figure 1 HEARTJNL2016309661F1:**
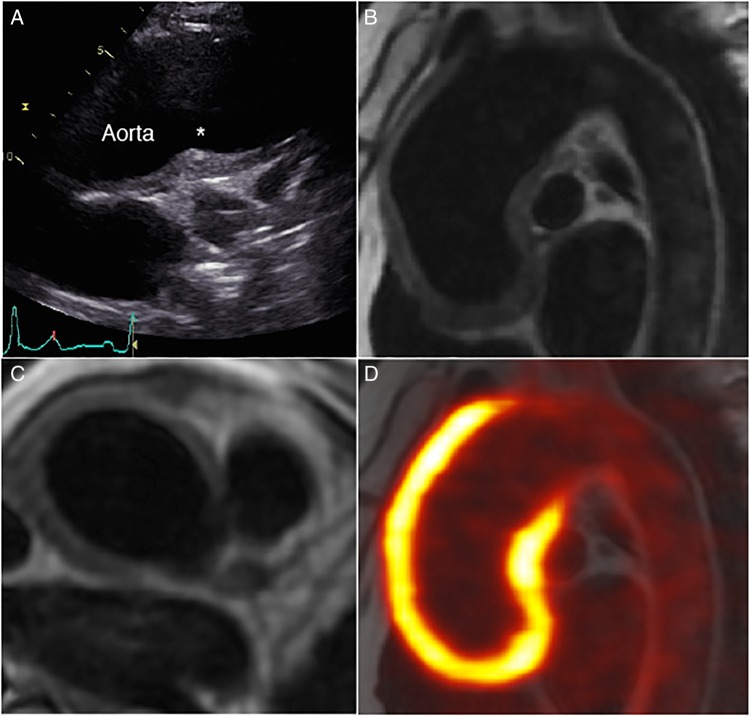
Transthoracic echocardiogram in high left parasternal long-axis view (A); cardiac MRI with black-blood sequence in sagittal (B) and axial planes (C); fused ^18^F-fluorodeoxyglucose positron emission tomography-MRI (D). The asterisk in 1A refers to para-aortic thickening.

## Answer: D

**Figure 2:**
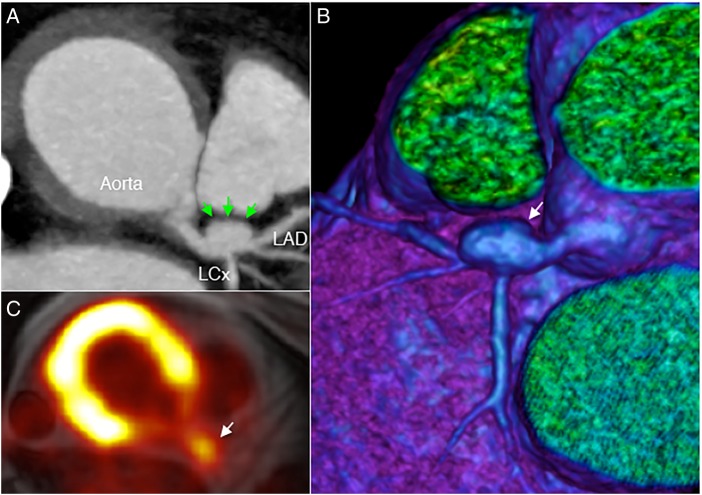
Coronary artery aneurysm (arrows) shown using CT angiography (A), three-dimensional CT reconstruction (B) and ^18^F-fluorodeoxyglucose positron emission tomography-MRI (C). LAD, left anterior descending artery; LCx, left circumflex artery.

Cardiac MRI shows marked circumferential proximal para-aortic soft tissue thickening, measuring 8.5 mm. Strikingly, a large (8 mm) coronary artery aneurysm involving the left main stem and proximal left anterior descending artery is also evident on MRI. The coronary artery aneurysm (arrows) was further characterised using CT angiography, shown in axial view and three-dimensional volume-rendered CT (figure 2). There was also a non-calcified atherosclerotic plaque at the transition of the aneurysmal and tubular left anterior descending artery, and bilateral subclavian stenoses. ^18^F-FDG PET images demonstrated severe inflammation of the proximal aorta (maximum standardised uptake value 6.7) and the coronary aneurysm. The underlying unifying diagnosis was vasculitis, most likely Takayasu's arteritis in type. While other vasculitides can cause aortitis, coronary involvement and presence of subclavian stenoses support the diagnosis of Takayasu's arteritis. This large-vessel granulomatous vasculitis typically affects the aorta and its main branches, causing stenosis at the vessel origin.^1^ Up to 40% of patients with Takayasu's arteritis also exhibit cardiac complications, which include aortic regurgitation, accelerated atherosclerosis, and, rarely, coronary artery aneurysm.^2^ Multi-modal cardiac imaging plays an important role in diagnosis and therapeutic monitoring.^3^ ^4^

## References

[1] Weyand CM, Goronzy JJ Medium- and large-vessel vasculitis. N Engl J Med 2003;349:160–9. 10.1056/NEJMra022694 12853590

[2] Kerr GS, Hallahan CW, Giordano J, et al Takayasu arteritis. Ann Intern Med 1994;120:919–29. 10.7326/0003-4819-120-11-199406010-00004 7909656

[3] Mason JC Takayasu arteritis--advances in diagnosis and management. Nat Rev Rheumatol 2010;6:406–15. 10.1038/nrrheum.2010.82 20596053

[4] Hartlage GR, Palios J, Barron BJ, et al Multimodality imaging of aortitis. JACC Cardiovasc Imaging 2014;7:605–19. 10.1016/j.jcmg.2014.04.002 24925329

